# Allelic Variations of the Waxy Gene and Their Associations with Indica–Japonica Differentiation and Amylose Content in Yunnan Local Rice Germplasm

**DOI:** 10.3390/genes16101198

**Published:** 2025-10-14

**Authors:** Ying Lv, Wei Deng, Xueqian Zuo, Duo Lan, Jing Tan, Jianhua Zhang, Yangjun Dong, Yuran Xu, Jinwen Zhang, Xiao Zhang, Jian Tu, Limei Kui, Anyu Gu, Xiqiong Shen, Xiaolin Li

**Affiliations:** 1 Food Crops Institute, Yunnan Academy of Agricultural Sciences, Kunming 650200, China; lvy@yaas.org.cn (Y.L.); nkylzsdw@yaas.org.cn (W.D.); zuoxueqian921@163.com (X.Z.); landuo@yaas.org.cn (D.L.); zhjhua6748@163.com (J.Z.); xuyuran@yaas.org.cn (Y.X.); zjwen@yaas.org.cn (J.Z.); tj@yaas.org.cn (J.T.); klm@yaas.org.cn (L.K.); ynzycxtd_gu@sina.com (A.G.); 15877998572@163.com (X.S.); 2 School of Agriculture, Yunnan University, Kunming 650504, China; tanjing@ynu.edu.cn; 3 Shuifu City Seed Promotion Station, Shuifu 657800, China; 13408867525@163.com (Y.D.); zx18708733431@163.com (X.Z.)

**Keywords:** Yunnan local germplasm, Waxy gene, Indica-japonica index, InDel markers, amylose content

## Abstract

Objectives: To provide insights for breeding high-quality rice varieties, we analyzed local rice (*Oryza sativa* L.) germplasm from Yunnan Province, China, focusing on the relationships among Waxy gene alleles, indica–japonica differentiation, and amylose content (AC). Methods: We examined 201 local rice accessions. Two functional molecular markers for the Waxy gene were used to detect four alleles (*Wx^a^*, *Wx^b^*, *Wx^in^*, *Wx^mw^*). Additionally, 33 InDel markers were employed to classify indica–japonica attributes, and AC was measured according to GB/T 15683-2008. Results: We detected 175 accessions with *Wx^a^*, 20 with *Wx^b^*, 4 with *Wx^in^*, and 2 with *Wx^mw^*, indicating *Wx^a^* dominance and a diverse genetic basis at the Waxy locus. Indica–japonica classification identified 180 indica-type, 19 japonica-type, and 2 intermediate-type accessions, confirming predominant indica differentiation in Yunnan rice. Integrating Waxy allele detection, indica–japonica attributes, and AC showed that *Wx^a^* occurred primarily in indica rice with higher AC (mean 22.55%), comparable to *Wx^in^* (mean 24.33%); *Wx^b^* was mainly found in japonica rice with lower AC (mean 13.46%), similar to *Wx^mw^* (mean 15.65%). Conclusions: Local Yunnan rice exhibits *Wx^a^* predominance at the Waxy locus and clear indica differentiation. The observed associations between Waxy alleles, subspecies attributes, and AC provide useful references for marker-assisted breeding of premium rice and for exploiting indica–japonica heterosis.

## 1. Introduction

Rice (*Oryza sativa*) is a cornerstone of global food security, serving as the primary staple for over 60% of the world’s population and contributing significantly to caloric intake in Asia [1]. The eating and cooking quality (ECQ) of rice grains, which encompasses traits like texture, stickiness, and flavor, is a critical factor influencing consumer preference and market value [2]. Within this, amylose content (AC) in the endosperm starch plays a pivotal role, determining gel consistency, retrogradation, and overall palatability [3]. High AC typically results in firmer, less sticky cooked rice, preferred in some cuisines, while low AC yields softer, glutinous textures [4].

The biosynthesis of amylose is primarily regulated by the *Waxy (Wx)* gene, located on chromosome 6, which encodes granule-bound starch synthase I (GBSSI) [5]. This enzyme catalyzes the elongation of amylose chains in the endosperm, and allelic variations at the *Wx* locus profoundly affect AC and ECQ [6]. The amylose content in rice endosperm is determined by the processing efficiency of *Wx* pre-mRNA, with the excision of intron I identified as the key regulatory step [7]. To date, at least nine *Wx* alleles have been identified, including *Wx^a^*, *Wx^b^*, *Wx^in^*, *Wx^mw^*, *Wx^lv^*, *Wx^la^*, *Wx^mq^*, *Wx^mp^*, and *Wx^op/hp^*, each arising from single nucleotide polymorphisms (SNPs), insertions/deletions (InDels), or splicing site mutations [8,9,10]. For instance, the G-to-T mutation at the 5′ splice site of intron 1 distinguishes *Wx^a^* (high AC, predominant in indica subspecies) from *Wx^b^* (low AC, common in japonica) by altering pre-mRNA splicing efficiency [11]. Further refinements include *Wx^in^*, derived from an A-to-C SNP in intron 6 on a *Wx^a^* background, which slightly reduces AC compared to *Wx^a^* [12], and *Wx^mw^*, a similar mutation on *Wx^b^* that maintains comparable low AC levels [13]. Recent studies have also uncovered ancestral alleles like *Wx^lv^*, which modulates grain mouthfeel by influencing amylose chain length [14], and *Wx^la^*, whose origin provides insights into domestication-driven quality improvements [15]. These variations not only explain up to 60–80% of AC phenotypic variance but also interact with other loci (e.g., ALK for alkali spreading) to fine-tune starch properties [16,17].

Beyond allelic diversity at Wx, rice germplasm exhibits profound subspecies differentiation between indica (*O. sativa* subsp. *indica*) and japonica (*O. sativa* subsp. *japonica*), shaped by independent domestication events, environmental adaptations, and human selection [18]. Indica varieties generally feature elongated grains, higher AC, and adaptation to tropical climates, while japonica types have rounder grains, lower AC, and tolerance to temperate conditions [19]. This differentiation manifests in genetic barriers, such as hybrid sterility in F1 generations, but also enables strong heterosis in inter-subspecific crosses, boosting yield by 15–30% [20]. Accurate classification of indica–japonica attributes is essential for exploiting this heterosis, and molecular markers like InDels have proven reliable, revealing fixed differences at thousands of loci [3,21].

Yunnan Province in southwestern China stands out as a global hotspot for rice genetic diversity and a putative center of Asian cultivated rice origin [8,10]. As one of the world’s major centers of genetic diversity, it harbors a wide range of unique landraces with distinct genetic backgrounds [22,23]. Its complex topography, ranging from low-altitude tropics to high-elevation plateaus, coupled with diverse climates and ethnic farming practices, has fostered extensive ecotypic variation [8]. Yunnan harbors both indica and japonica landraces, along with wild relatives (e.g., *O. rufipogon*), and boasts over 58 cultivated variants [24]. Studies using SSR and SNP markers have documented high nucleotide diversity (π) in Yunnan rice, with indica–japonica differentiation varying along altitudinal gradients—indica dominating lowlands and japonica higher elevations [25]. A systematic characterization of Waxy (Wx) allele distribution and its relationship with amylose content and subspecies differentiation in this region is essential for germplasm conservation, breeding strategy optimization, and for complementing global rice genetic studies. This richness provides untapped potential for quality breeding, yet comprehensive analyses linking Wx alleles, subspecies attributes, and AC in local germplasm remain limited [26]. Current research on rice quality often examines the Wx gene, subspecies identity (indica/japonica), and amylose content (AC) in isolation, rather than as an integrated system [2,16]. The interaction between functional Wx alleles and their genetic backgrounds remains poorly understood, particularly in diverse germplasm. This disconnect is evident in regions like Yunnan, where rich genetic resources coexist with significant indica–japonica differentiation. The lack of combined analysis limits the understanding of how subspecies background modulates the effect of Wx alleles on AC. Such improvement is essential for effective marker-assisted breeding aimed at improving rice quality through informed utilization of genetic diversity.

Therefore, in this study, we designed experiments with the following specific objectives: genotyped 201 Yunnan local rice accessions using two functional markers to detect *Wx^a^*, *Wx^b^*, *Wx^in^*, and *Wx^mw^* alleles, classified indica–japonica attributes with 33 InDel markers, and quantified AC per national standards. This study aimed to identify key allelic distributions linked to subspecies differentiation and AC, to support practical marker-assisted selection (MAS) and the development of high-quality, high-yielding rice varieties.

## 2. Materials and Methods

### 2.1. Plant Materials

A total of 201 local rice accessions from various Yunnan regions were sourced from the Grain Crops Research Institute, Yunnan Academy of Agricultural Sciences (Table S1).

### 2.2. DNA Extraction

Genomic DNA was extracted from 3–4 leaf-stage seedlings using a modified rapid method: 200 μL extraction buffer (100 mmol/L Tris-HCl pH 8.7, 1 mol/L KCl, 10 mmol/L EDTA; 1:1:1 ratio) per sample, autoclaved at 101 °C for 10 min, diluted to 25 ng/μL with deionized water, and stored at −20 °C. DNA quantity and quality were evaluated using a NanoDrop 2000 spectrophotometer (Thermo Fisher Scientific, Wilmington, DE, USA).

### 2.3. Waxy Gene Genotyping

Two functional markers, Waxygt-ARMS and Waxyac-ARMS [10], were used (Table S2). To ensure robustness and reproducibility, preliminary optimization was performed including gradient PCR (annealing temperatures from 55 °C to 65 °C), testing of primer ratios (0.2–1.0 μM), and titration of DNA polymerase concentration. PCR reactions (10 μL) contained 5 μL 2× Taq Master Mix (Dye) (Xuzhou Probe Gene Technology Co., Ltd. No. 99 Daxue Road, Xuzhou City, Jiangsu, China), 1 μL primers (inner:outer ratio 1.5:1), 1 μL template DNA, and 3 μL ddH_2_O. Cycling: 95 °C for 5 min; 30 cycles of 95 °C for 30 s, 56 °C for 30 s, 72 °C for 40 s; 72 °C for 10 min. Products were separated on 8% denaturing polyacrylamide gels and silver-stained. Band patterns identified alleles: *Wx^a^* (287/143 bp, 185/113 bp); *Wx^b^* (297/198 bp, 185/113 bp); *Wx^in^* (287/143 bp, 185/126 bp); *Wx^mw^* (297/198 bp, 185/126 bp).

### 2.4. Indica–Japonica Classification

Thirty-three InDel markers [11] were used (Table S3). PCR conditions mirrored Waxy genotyping but without inner/outer primers; annealing temperatures varied per marker. Bands were compared to standards (9311 for indica, Nipponbare for japonica). Indica index (*F*_A_) and Japonica index (*F*_B_) were calculated as:

$$Indica\;index\left(F_{A}\right)=\frac{2\sum_{1}^{N}X_{A}+\sum_{1}^{N}X_{H}}{2N-2X_{-}}$$

$$Japonica\;index\left(F_{B}\right)=\frac{2\sum_{1}^{N}X_{B}+X_{H}}{2N-2X_{-}}$$

where *X_A_*, *X_H_*, *X_B_* are homozygous indica, heterozygous, and homozygous japonica loci; *N* is total loci. Classification followed Table S4.

### 2.5. Amylose Content Measurement

The amylose content of rice grain samples was determined using the iodine colorimetric method as specified in GB/T 15683-2025 [27]. Rice grains were ground into a fine powder, passed through a 0.5 mm sieve, and dried at 40 °C to constant weight. A 100 mg sample (weighed to 0.1 mg accuracy) was dispersed in 1 mL of 95% ethanol and 9 mL of 1 mol/L NaOH in a 100 mL volumetric flask. The mixture was heated in a boiling water bath for 10 min to gelatinize the starch, cooled to room temperature, and diluted to 100 mL with distilled water. A 5 mL aliquot was transferred to a 50 mL volumetric flask, neutralized with 1 mL of 1 mol/L acetic acid, and mixed with 2 mL of iodine solution (0.2% I2 in 2% KI). The solution was diluted to 50 mL with distilled water and allowed to stand for 20 min to develop a stable blue color. Absorbance was measured at 620 nm using a spectrophotometer, with distilled water as the blank. Each sample was analyzed in three technical replicates. A standard curve, prepared with known amylose standards, was used to calculate the amylose content (% dry weight). For detailed procedures, including reagent preparation and calculation formulas, refer to GB/T 15683-2025 (https://openstd.samr.gov.cn/, accessed on 1 August 2025).

## 3. Results

### 3.1. Geographic and Ecological Diversity of Sampled Germplasm

**Figure 1:**
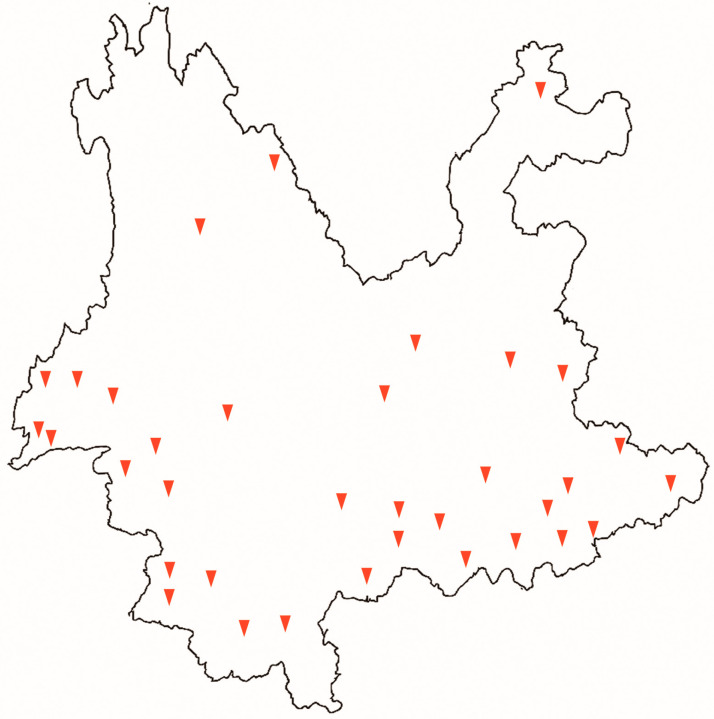
Sampling map of Yunnan Province depicting the distribution of sampled counties, derived from administrative boundary data provided by the National Geomatics Center of China under map review number YunS(2021)46 (http://bzdt.ch.mnr.gov.cn/, accessed on 20 July 2025). Counties with sampling sites are marked by red inverted triangles.

Of the 201 rice accessions, 179 were sourced from 35 counties across 12 of Yunnan’s 16 prefecture-level administrative divisions, encompassing approximately 27% of the province’s 129 counties and achieving a coverage rate of 75% at the prefectural level. The remaining 22 accessions lacked confirmed sources of origin. This sampling strategy captures the province’s complex topography and climatic variability, spanning from high-altitude plateaus in the northwest, such as Ninglang County (elevations exceeding 3000 m), to low-elevation tropical regions in the southeast, like Jinghong County in Xishuangbanna Prefecture (average elevation around 550 m). The sampled counties include highland areas (e.g., Ninglang and Jianchuan Counties), mountainous terrains (e.g., Yun and Yongde Counties), and river valleys (e.g., Yuanyang and Jinghong Counties), representing diverse ecosystems from subtropical forests to alpine meadows and supporting a wide array of agricultural environments that enhance the representativeness of the germplasm for studying rice genetic diversity (Figure 1, Table 1).

### 3.2. Allelic Variation Statistics of the Waxy Gene in 201 Yunnan Local Rice Germplasm Accessions

**Figure 2:**
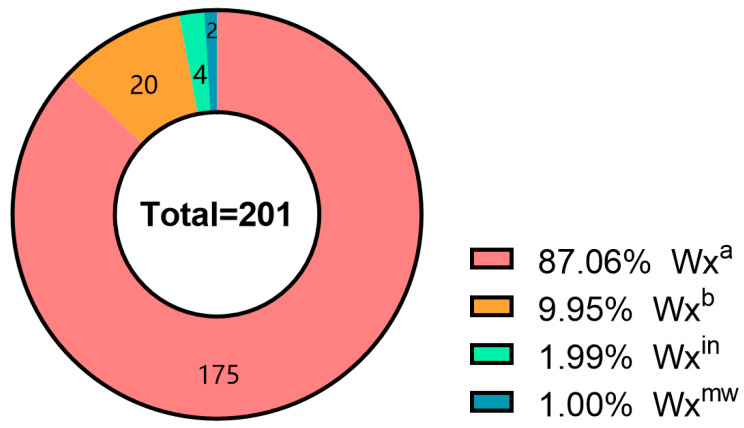
Allelic Variation Statistics of the Waxy Gene in 201 Yunnan Local Rice Germplasm Accessions. Four colors represent distinct genotypes: *Wx^a^* (*n* = 175), *Wx^b^* (*n* = 20), *Wx^in^* (*n* = 4), and *Wx^mw^* (*n* = 2).

Waxy^gt^-ARMS amplified 287/143 bp in 180 accessions (G allele) and 297/198 bp in 21 (T allele) (Figure 2). Waxy^ac^-ARMS yielded 185/113 bp in 195 (A allele) and 185/126 bp in 6 (C allele). Combined, alleles were: 175 *Wx^a^*, 20 *Wx^b^*, 4 *Wx^in^*, 2 *Wx^mw^* (Figure 2, Table S5).

### 3.3. Distribution of Indica and Japonica Characteristics Among Yunnan Local Rice Germplasm Accessions with Different Waxy Genotypes

**Figure 3:**
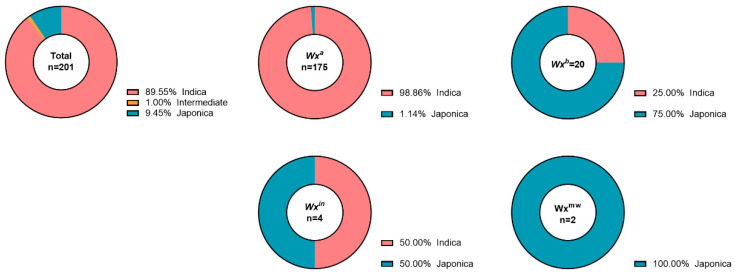
Distribution of Indica and Japonica Characteristics among Yunnan Local Rice Germplasm Accessions with Different Waxy Genotypes. Indica categories include Typical Indica, Indica, and Indica-Leaning; Japonica categories include Japonica-Leaning, Japonica, and Typical Japonica.

InDel markers classified 180 indica (121 typical indica, 53 indica, 6 indica-leaning), 2 intermediate, 19 japonica (2 japonica-leaning, 9 japonica, and 8 typical japonica). *Wx^a^* occurred in 175 indica types; *Wx^b^* in 15 japonica and 5 indica; *Wx^in^* in 2 indica and 2 japonica; *Wx^mw^* in 2 japonica. A chi-square test showed a highly significant association between Waxy genotype and rice type (χ^2^ = 197.3, df = 18, *p* < 0.001). *Wx^a^* was mainly found in Indica (95.4%, including both typical indca and indca), while *Wx^b^* was more common in Japonica (75.0%, including both typical japonica and japonica), indicating a strong correlation between Waxy genotype and rice type (Figure 3, Table 2 and Table S6).

### 3.4. Statistical Analysis Between Different Waxy Genotypes and Their Amylose Content

**Figure 4:**
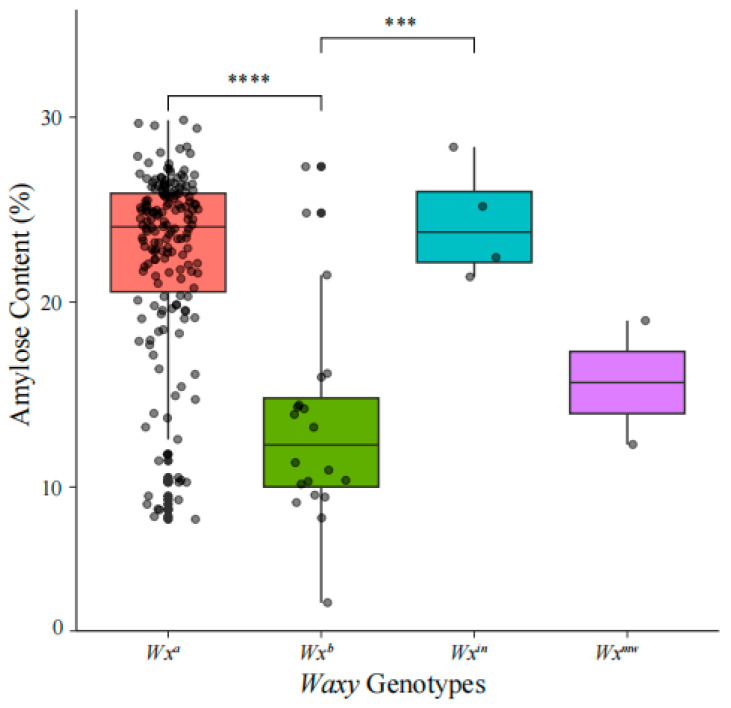
Significant differences among genotypes were assessed by one-way ANOVA followed by Tukey’s HSD test (*** *p* < 0.001; **** *p* < 0.0001).

Amylose content was determined for 201 Yunnan local rice germplasm accessions, revealing significant variation across different Waxy genotypes. AC ranged from 3.74% (Ma Xian Gu(2), ID 139, *Wx^b^*) to 29.84% (Chi Bai Gu, ID 27, *Wx^a^*), with an overall mean of 21.70% (standard deviation: 5.74%). Among the genotypes, *Wx^a^* (175 accessions) exhibited the highest average AC at 22.55% (range: 8.26–29.84%), followed by *Wx^in^* (4 accessions) at 24.33% (range: 21.36–28.39%). In contrast, *Wx^b^* (20 accessions) and *Wx^mw^* (2 accessions) showed lower mean AC values of 13.46% (range: 3.74–24.82%) and 15.65% (range: 12.30–19.00%), respectively. Statistical analysis confirmed that accessions with *Wx^a^* and *Wx^in^* genotypes had significantly higher AC compared to those with *Wx^b^* (Figure 4, Table S7).

## 4. Discussion

Our analysis of 201 Yunnan local rice accessions revealed a diverse yet *Wx^a^*-dominant allelic landscape at the Waxy locus, with 175 accessions carrying *Wx^a^* (87.06%), 20 *Wx^b^* (9.95%), 4 *Wx^in^* (1.99%), and 2 *Wx^mw^* (1.00%). This predominance of *Wx^a^* aligns with global patterns in indica-heavy germplasm [6,14] and underscores Yunnan’s role as an indica-rich diversity center [28]. The detection of rarer alleles like *Wx^in^* and *Wx^mw^*, though limited in number, highlights untapped variation potentially useful for fine-tuning AC [12,13]. Methodologically, the ARMS-PCR approach proved efficient for multiplex allele discrimination, confirming band patterns consistent with prior validations [11]. However, occasional non-specific bands suggest optimizations in primer ratios or enzyme activity could enhance robustness [17].

Integrating Wx genotyping with AC measurements demonstrated clear allele-specific effects: *Wx^a^* and *Wx^in^* accessions exhibited high mean AC (22.55% and 24.33%, respectively), while *Wx^b^* and *Wx^mw^* showed lower values (13.46% and 15.65%). These findings corroborate literature indicating *Wx^a^* drives high amylose synthesis via efficient splicing, resulting in firmer grains [3,11], whereas *Wx^b^* reduces it through splicing defects [4]. The comparable AC between *Wx^in^* and *Wx^a^* supports subtle modulatory roles of the intron 6 SNP [12], and similarly for *Wx^mw^* on *Wx^b^* [13]. Intriguingly, our data did not fully replicate reports of *Wx^in^* elevating AC on *Wx^a^* backgrounds or lowering it on *Wx^b^* [6], possibly due to small sample sizes for rare alleles or interactions with background loci like ALK or SSIIa [15,16]. Future studies with sufficiently large sample sizes should be conducted using balanced designs to validate these findings. In addition, potential interactions between *ALK* and *SSIIa* alleles should be carefully examined to clarify their confounding effects. Broader comparisons reveal parallels with Korean rice collections, where Wx haplotypes explained ECQ variance [20], and Vietnamese cultivars, where SNPs at Wx modulated glycemic index via AC [21]. In Yunnan context, these alleles may interact with environmental factors (e.g., altitude), as high-elevation landraces often exhibit altered starch profiles [10].

It should be specifically noted that the apparent amylose content of 3.74% measured by iodine colorimetry in this study may have limitations. This value could partially originate from non-specific binding of iodine to long-chain amylopectin rather than true amylose, a phenomenon particularly significant in low-amylose samples [1]. Although the iodine colorimetric method offers advantages of operational simplicity and low cost, its accuracy is highly dependent on the quality of standard curve construction [2]. When using potato amylose for calibration, the iodine-binding capacity of amylopectin may lead to false positive values in waxy rice samples [3]. In contrast, physical analysis methods such as Size Exclusion Chromatography (SEC) and Differential Scanning Calorimetry (DSC) can directly resolve starch molecular structure, providing more accurate results for waxy samples [4]. While the standard curve in this study was strictly prepared according to GB/T 15683-2008, future research will consider adopting mixed standard (amylose-amylopectin) calibration curves to further improve detection accuracy in the low concentration range [29].

Subspecies classification via InDel markers identified 180 indica-types (89.55%), 19 japonica-types (9.45%), and 2 intermediates (1.00%), confirming pronounced indica–japonica differentiation with indica dominance [8,30]. This mirrors altitudinal patterns in Yunnan, where indica prevails in warmer lowlands and japonica in cooler highlands [31]. *Wx^a^* was overwhelmingly associated with indica (173/175), reinforcing subspecies-specific distributions [6,18], while *Wx^b^* appeared in 15 japonica but also 5 indica, suggesting occasional introgression [28]. Rare alleles like *Wx^in^* spanned both subspecies, hinting at ancestral polymorphisms predating differentiation [14,15]. The low frequency of intermediates (1%) contrasts with some Yunnan studies reporting higher admixture [32], potentially due to our germplasm’s bias toward cultivated landraces. Nonetheless, these intermediates could facilitate indica–japonica hybrids, enhancing fertility and yield via heterosis [19,20].

From a breeding perspective, our results advocate MAS using Wx functional markers to tailor AC for premium varieties—e.g., incorporating *Wx^in^* into *Wx^a^* backgrounds for moderate AC and improved palatability [14]. The identified *Wx^in^* and *Wx^mw^* donors could adjust starch viscosity and fissure resistance [16], addressing quality issues in high-yielding hybrids. Exploiting Yunnan’s diversity for heterosis, as in indica–japonica crosses, may boost grain quality traits [33], especially with intermediates bridging compatibility [34]. Limitations include the focus on four alleles; future work should screen for emerging variants like *Wx^lv^* or *Wx^la^* using whole-genome sequencing to capture epistatic interactions [35].

In this study, although our findings are consistent with previously reported associations between Waxy (Wx) alleles, amylose content, and indica–japonica differentiation, the work provides an important regional contribution. The predominance of the *Wx^a^* allele (87.06%) in Yunnan aligns with its high frequency in indica-rich regions like Vietnam (83.5%) but contrasts sharply with temperate japonica collections from Korea where *Wx^b^* predominates (91.3%) [14]. Yunnan Province is recognized as one of the global centers of rice genetic diversity, harboring a wide range of landraces with unique genetic backgrounds. Systematically characterizing the distribution of Wx alleles in this germplasm not only confirms global patterns but also offers critical baseline data for future research and breeding [22,23].

Unlike many previous studies that focused primarily on identifying novel Wx alleles, our analysis emphasizes the allele frequency distribution and its association with subspecies identity in a large, representative set of Yunnan rice germplasm. This provides valuable information for germplasm conservation, selection of parental lines for breeding, and for monitoring allele shifts under modern breeding and climate change. Even though no completely new alleles were detected, these findings strengthen the understanding of the genetic structure of local rice populations and complement global research on rice quality improvement.

In conclusion, this study elucidates Wx allelic dynamics in Yunnan rice, linking them to subspecies attributes and AC. These insights pave the way for targeted breeding, leveraging local germplasm to develop high-quality, resilient varieties amid climate challenges.

## 5. Conclusions

Yunnan rice exhibits Waxy allelic diversity with *Wx^a^* predominance, linked to indica types and higher AC. These insights facilitate breeding for quality and heterosis utilization.

## Figures and Tables

**Table 1:** Geographic origin and ecological distribution of the 201 Yunnan local rice accessions.

ID	Variety Count	Origin	Ecological Type
1	4	Ninglang City	Low-latitude plateau climate
2	5	Jianchuan County	Low-latitude plateau monsoon climate
3	10	Yingjiang County	Subtropical plateau monsoon climate
4	8	Lianghe County	South subtropical monsoon climate
5	3	Longchuan County	Subtropical monsoon climate
6	2	Ruili City	South subtropical monsoon climate
7	11	Longling County	Low-latitude monsoon climate
8	2	Zhenkang County	Subtropical monsoon climate
9	9	Yongde County	Subtropical monsoon climate
10	6	Yun County	Subtropical monsoon climate
11	3	Gengma County	Subtropical monsoon climate
12	5	Ximeng Va Nationality Autonomous County	Subtropical monsoon climate
13	6	Menglian Daizu Lahuzu Wazu Autonomous County	South subtropical monsoon climate
14	13	Lancang County	Subtropical montane monsoon climate with wet summers and dry winters
15	5	Menghai County	Tropical and subtropical southwest monsoon climate
16	4	Jinghong City	Tropical rainforest climate
17	12	Jiangcheng County	Subtropical monsoon climate
18	28	Mojiang Hani Autonomous County	Subtropical monsoon climate
19	7	Lvchun County	Subtropical montane monsoon climate
20	5	Honghe County	Tropical and subtropical monsoon climate
21	7	Yuanyang County	Subtropical montane monsoon climate
22	4	Jinping County	Tropical rainforest climate
23	3	Kaiyuan City	Subtropical monsoon climate
24	2	Pingbian County	Subtropical montane monsoon climate
25	3	Wenshan City	Mid-subtropical monsoon climate
26	2	Maguan County	Subtropical monsoon climate
27	1	Malipo County	Subtropical monsoon climate
28	1	Yanshan County	Subtropical monsoon climate
29	1	Funing County	South subtropical monsoon climate
30	11	Guangnan County	Mid-subtropical plateau monsoon climate
31	1	Luoping County	South subtropical climate and plateau monsoon climate
32	3	Luliang County	Subtropical plateau monsoon climate
33	5	Yimen County	Subtropical monsoon climate
34	6	Fumin County	Low-latitude subtropical plateau monsoon climate
35	3	Yanjin County	Mid-subtropical and temperate monsoon climates

**Table 2:** Analysis of the Association between Waxy Genotype and Indica–Japonica type.

Waxy Genotype	Typical Indica (*n*, %)	Indica Rice (*n*, %)	Leaning Indica (*n*, %)	Intermediate Type (*n*, %)	Leaning Japonica (*n*, %)	Japonica Rice (*n*, %)	Typical Japonica (*n*, %)	χ^2^ Contribution
*Wx^a^*	116 (66.3%)	51 (29.1%)	6 (3.4%)	2 (1.1%)	0 (0.0%)	0 (0.0%)	0 (0.0%)	136.0
*Wx^b^*	4 (20.0%)	1 (5.0%)	0 (0.0%)	0 (0.0%)	2(10.0%)	7 (35.0%)	6 (30.0%)	46.4
*Wx^in^*	1 (25.0%)	1 (25.0%)	0 (0.0%)	0 (0.0%)	0 (0.0%)	0 (0.0%)	2 (50.0%)	7.4
*Wx^mw^*	0 (0.0%)	0 (0.0%)	0 (0.0%)	0 (0.0%)	0 (0.0%)	2 (100.0%)	0 (0.0%)	7.5
Total	121 (60.2%)	53 (26.4%)	6 (3.0%)	2 (1.0%)	2 (1.0%)	9 (4.5%)	8(4.0%)	χ^2^ = 197.3 *

* Values represent *n* (%). The χ^2^ contribution per cell was computed as (O–E)^2^/E, and the total χ^2^ statistic is the sum of individual contributions.

## Data Availability

The original contributions presented in the study are included in the article/Supplementary Material, further inquiries can be directed to the corresponding author.

## Supplementary Materials

The following supporting information can be downloaded at: https://www.mdpi.com/article/10.3390/genes16101198/s1, Table S1. Sources of 201 Yunnan Local Rice Germplasm Accessions. Table S2. Information of molecular markers of Waxy gene alleles. Table S3. Information of molecular markers of Indica-Japonica Classification. Table S4. Criteria for Classification of Indica and Japonica Rice. Table S5. Details of Allelic Variations in the Waxy Gene across 201 Yunnan Local Rice Germplasm Accessions. Table S6. Amylose Content Associated with Different Waxy Genotypes in 201 Yunnan Local Rice Germplasm Accessions. Table S7. Indica-Japonica Attributes Associated with Different Waxy Genotypes in 201 Yunnan Local Rice Germplasm Accessions.

## Abbreviations

AC	Amylose content
ALK	Gene associated with alkali spreading value/gelatinization temperature (linked with SSIIa)
ARMS-PCR	Amplification Refractory Mutation System PCR
bp	Base pair
ECQ	Eating and cooking quality
FA	Indica index
FB	Japonica index
GBSSI	Granule-bound starch synthase I
InDel	Insertion–deletion polymorphism
MAS	Marker-assisted selection
PCR	Polymerase chain reaction
SD	Standard deviation
SNP	Single-nucleotide polymorphism
SSR	Simple sequence repeat
SSIIa	Soluble starch synthase IIa
Wx	*Waxy* gene (granule-bound starch synthase locus)
