# Supplementary material for: Allelic Variations of the Waxy Gene and Their Associations with Indica–Japonica Differentiation and Amylose Content in Yunnan Local Rice Germplasm
Source: Genes (Basel). 2025 Oct 14;16(10):1198. doi: 10.3390/genes16101198 (PMC12564750; doi:10.3390/genes16101198)
Supplement: Supplementary file 1 [file genes-16-01198-s001.zip › genes-3859168-supplementary.pdf]

# Supplementary Materials

*Article*

# Allelic Variations of the Waxy Gene and Their Associations with Indica–Japonica Differentiation and Amylose Content in Yunnan Local Rice Germplasm

**Ying Lv [1,†], Wei Deng [1,†], Xueqian Zuo [1], Duo Lan [1], Jing Tan [2], Jianhua Zhang [1], Yangjun Dong [3], Yuran Xu [1], Jinwen Zhang [1], Xiao Zhang [3], Jian Tu [1], Limei Kui [1], Anyu Gu [1], Xiqiong Shen [1] and Xiaolin Li [1,*]**

[1] Food Crops Institute, Yunnan Academy of Agricultural Sciences, Kunming 650200, China; lvy@yaas.org.cn (Y.L.); nkylzsdw@yaas.org.cn (W.D.); zuoxueqian921@163.com (X.Z.); landuo@yaas.org.cn (D.L.); zhjhua6748@163.com (J.Z.); xuyuran@yaas.org.cn (Y.X.); zjwen@yaas.org.cn (J.Z.); tj@yaas.org.cn (J.T.); klm@yaas.org.cn (L.K.); ynzycxtd_gu@sina.com (A.G.); 15877998572@163.com (X.S.)
[2] School of Agriculture, Yunnan University, Kunming 650504, China; tanjing@ynu.edu.cn
[3] Shuifu City Seed Promotion Station, Shuifu 657800, China; 13408867525@163.com (Y.D.); zx18708733431@163.com (X.Z.)
* Correspondence: lixiaolin@yaas.org.cn
† These authors contributed equally to this work.

**Table S1.** Sources of 201 Yunnan Local Rice Germplasm Accessions.

| ID | Variety | Origin |
|---|---|---|
| 1 | Ba Bao Gu | Guangnan |
| 2 | Ba Qi | Longling |
| 3 | Ba Yue Hong Gu | Pingbian |
| 4 | Ba Yue Nuo | Menglian |
| 5 | Bai Gan Hong Gu(1)* | Longling |
| 6 | Bai Gan Hong Gu(2)* | Longling |
| 7 | Bai Gan Lu Feng | Longling |
| 8 | Bai Gen Qi Tou | Menglian |
| 9 | Bai Gu | Menghai |
| 10 | Bai Gu Hong Mi | Wenshan |
| 11 | Bai Hua Pi Nuo | Yongde |
| 12 | Bai Liang Gu | Ynajin |
| 13 | Bai Mao Xiang Gu(1)* | Yongde |
| 14 | Bai Mao Xiang Gu(2)* | Yongde |
| 15 | Bai Mian Gu | Mojiang |
| 16 | Bai Pi Hong Mi Ma Zha Gu | Jiangcheng |
| 17 | Bai Xiang Gu | Kaiyuan |
| 18 | Bai Xiang Nuo | Pingbian |
| 19 | Bai Xiang Nuo Gu | Jianchuan |
| 20 | Ban He Yi Hao Gu | Jiangcheng |
| 21 | Ban Jie Mang | Luliang |

| ID | Variety | Origin |
|---|---|---|
| 22 | Bi Wu Sheng | Yingjiang |
| 23 | Bian Liang Gu | Ximeng |
| 24 | Bie Wu Sheng | Jianchuan |
| 25 | Bo Li Nuo | Mojiang |
| 26 | Cheng Du Ai | Lancang |
| 27 | Chi Bai Gu | Lancang |
| 28 | Chong Tui(1)* | Ninglang |
| 29 | Chong Tui(2)* | Ninglang |
| 30 | Chu Ya Gu | Fumin |
| 31 | Da Ai Ke | Mojiang |
| 32 | Da Bai Gu(1)* | Ximeng |
| 33 | Da Bai Gu(2)* | Lancang |
| 34 | Da Bai Gu(3)* | Menglian |
| 35 | Da Bai Nuo(1)* | Lancang |
| 36 | Da Bai Nuo(2)* | Jinghong |
| 37 | Da Er Xiang | Wenshan |
| 38 | Da Hei Gu | Fumin |
| 39 | Da Hei Ke Gu | Mojiang |
| 40 | Da Hong Gu(1)* | Lancang |
| 41 | Da Hong Gu(2)* | Jinghong |
| 42 | Da Hong Gu(3)* | Yunxian |
| 43 | Da Hong Pi Nuo | Zhenkang |
| 44 | Da Hua Nuo | Yuanyang |
| 45 | Da Huang Pi Nuo | Lancang |
| 46 | Da Li Gu | Yimen |
| 47 | Da Long Gu | Mojiang |
| 48 | Da Luo Ping | Mojiang |
| 49 | Da Ma Zha Gu | Malipo |
| 50 | Da Qian Qian Gu | Yanjin |
| 51 | Da Sha Ren Gu | Gengma |
| 52 | Da Wa Gu | Lianghe |
| 53 | Da Zi Gan | Longling |
| 54 | Ding Liang Gu | Jianchuan |
| 55 | Dong Gan Wu Ju | Yuanyang |
| 56 | Dong Li Hong Gu | Maguan |
| 57 | Duan Jiao Bai Gu | Yongde |
| 58 | Er Bai Diao | Longling |
| 59 | Er Bai Gu | Jianchuan |
| 60 | Er Kuai Gu | Mojiang |
| 61 | Gan Zha Gu | Longling |
| 62 | Gao Liang Nuo | Yimen |
| 63 | Gao San Sui | Longling |
| 64 | Gao Tai Ba Hao | Guangnan |

| ID | Variety | Origin |
|---|---|---|
| 65 | Guang Nan Gu | Guangnan |
| 66 | Guangnan Yi Hao | Guangnan |
| 67 | Hai Gu | Yimen |
| 68 | Han Luo Ping | Mojiang |
| 69 | Hao An Wang | Ruili |
| 70 | Hao An Wang Mie Duan | Yingjiang |
| 71 | Hao Ba Wan | Longchuan |
| 72 | Hao Bu Ka | Jinghong |
| 73 | Hao Dong Nan | Menghai |
| 74 | Hao Gan | Menglian |
| 75 | Hao Ge Lao | Yingjiang |
| 76 | Hao Hao Nen | Yingjiang |
| 77 | Hao Hua Xiang | Yingjiang |
| 78 | Hao Jiang Duo | Yingjiang |
| 79 | Hao Ma Liang | Yingjiang |
| 80 | Hao Men Lia | Yingjiang |
| 81 | Hao Mie Han | Gengma |
| 82 | Hao Mu Lei | Lianghe |
| 83 | Hao Mu Xi(1)* | Lianghe |
| 84 | Hao Mu Xi(2)* | Yingjiang |
| 85 | Hao Yu Bang Nan | Menghai |
| 86 | Hao Zao Hao | Longchuan |
| 87 | Hei Zi Gan | Longling |
| 88 | Hei Zi Mi | Lvchun |
| 89 | Hong Gan Ma Zha Gu | Guangna |
| 90 | Hong Gu(1)* | Jinghong |
| 91 | Hong Gu(2)* | Ruili |
| 92 | Hong Gu(3)* | Yunxian |
| 93 | Hong Han Nuo | Yunxian |
| 94 | Hong He Hong Mi | Honghe |
| 95 | Hong Hua Gu | Kaiyuan |
| 96 | Hong Hua Mi | Yuanyang |
| 97 | Hong Jiao Bai | Jinping |
| 98 | Hong Mi | Lvchun |
| 99 | Hong Mi Ma Zhan | Ninglang |
| 100 | Hong Pi Xiang | Kaiyuan |
| 101 | Hong Xiang Gu(1)* | Yongde |
| 102 | Hong Xiang Gu(2)* | Yongde |
| 103 | Hong Yun Dang | Lianghe |
| 104 | Hong Zao Gu | Yuanyang |
| 105 | Hua Er Zao | Longling |
| 106 | Hua Gu San Bai Bang | Guangnan |
| 107 | Hua Pi Hong Gu | Yunxian |

| ID | Variety | Origin |
| --- | --- | --- |
| 108 | Huang Ban Suo | Lianghe |
| 109 | Huang Hui Xiang | Luoping |
| 110 | Huang Pi Ruan Mi | Lancang |
| 111 | Huang Pi Xiang | Yimen |
| 112 | Huang Xiang Nuo | Mojiang |
| 113 | Huang Xin Nuo | Lianghe |
| 114 | Ji You Nuo | Luliang |
| 115 | Jia Xiang | Luliang |
| 116 | Jian Tang Zao | Guangnan |
| 117 | Jie Gu Nuo(1)* | Lancang |
| 118 | Jie Gu Nuo(2)* | Jiangcheng |
| 119 | Jie Gu Nuo(3)* | Ximeng |
| 120 | Jin Bao Yin | Wenshan |
| 121 | Jiu Yue Gu | Jiangcheng |
| 122 | Jiu Yue Nuo | Lvchun |
| 123 | Kun Ming Xiao Bai Gu | Mojiang |
| 124 | Lan Pi Gu | Mojiang |
| 125 | Lao Gong Gu | Mojiang |
| 126 | Lao Gua Gu | Mojiang |
| 127 | Lao Xiang Gu | Mojiang |
| 128 | Lao Yang Gu | Mojiang |
| 129 | Leng Shui Bai Gu | Zhenkang |
| 130 | Long Gu | Funing |
| 131 | Lu Shui Nuo | Yunxian |
| 132 | Luan Jiao Bai Gu | Mojiang |
| 133 | Luan Jiao Diao | Funing |
| 134 | Lue Ha | Lvchun |
| 135 | Luo Ping Gu | Ximeng |
| 136 | Ma Deng Hong Gu | Jianchuan |
| 137 | Ma Ti Gu | Mojiang |
| 138 | Ma Xian Gu(1)* | Lancang |
| 139 | Ma Xian Gu(2)* | Yunxian |
| 140 | Ma Zha Gu(1)* | Menghai |
| 141 | Ma Zha Gu(2)* | Guangnan |
| 142 | Ma Zha Gu(3)* | Jinping |
| 143 | Ma Zha Gu(4)* | Maguan |
| 144 | Ma Zha Gu(5)* | Jinping |
| 145 | Ma Zha Nuo | Guangnan |
| 146 | Mang Shi Gu | Yongde |
| 147 | Mang Zhong Gu | Yimen |
| 148 | Mao Ke Hong Nuo | Guangnan |
| 149 | Mao Xiang Gu | Guangnan |
| 150 | Mao Xiang Nuo | Yingjiang |

| ID | Variety | Origin |
|---|---|---|
| 151 | Meng Ka Zha | Jiangcheng |
| 152 | Meng Lu Gu | Jiangcheng |
| 153 | Meng Wang Gu(1)* | Jiangcheng |
| 154 | Meng Wang Gu(2)* | Mojiang |
| 155 | Meng Xian Da Bai Gu | Jiangcheng |
| 156 | Mo Wang Gu | Lancang |
| 157 | Niao Ju Qi Tou | Mojiang |
| 158 | Niu Yang Gu | Jiangcheng |
| 159 | Ou Yang Gu | Yongde |
| 160 | Qi Tou Gu | Menghai |
| 161 | Qi Yue Nuo | Yuanyang |
| 162 | Qing Ming Li Hong Gu | Honghe |
| 163 | Shan Dao Hong Gu | Mojiang |
| 164 | Ta Pi Gu | Yuanyang |
| 165 | Tu Po Huo Lue | Lvchun |
| 166 | Tuan Ke Li | Longling |
| 167 | Wu Bai Li | Yanjin |
| 168 | Wu Ju Gu | Mojiang |
| 169 | Xi Bai Gu(1)* | Lancang |
| 170 | Xi Bai Gu(2)* | Ximeng |
| 171 | Xi Gan Qi Tou | Menglian |
| 172 | Xi Luo Ping | Mojiang |
| 173 | Xi Ma Xian Gu | Jiangcheng |
| 174 | Xi Ma Zha Gu | Mojiang |
| 175 | Xiang Diao | Fumin |
| 176 | Xiang Gu(1)* | Lianghe |
| 177 | Xiang Gu(2)* | Longchuan |
| 178 | Xiang Gu(3)* | Jinping |
| 179 | Xiang Nuo | Menglian |
| 180 | Xiao Bai Gu | Lancang |
| 181 | Xiao Bai Nuo | Lianghe |
| 182 | Xiao Hong Gu(1)* | Lancang |
| 183 | Xiao Hong Gu(2)* | Yanshan |
| 184 | Xiao Hong Gu(3)* | Lvchun |
| 185 | Xiao Luo Ping | Mojiang |
| 186 | Xiao Ma Zha Gu | Honghe |
| 187 | Xiao Wu Ju Gu | Jiangcheng |
| 188 | Xiao Zi Gan | Mojiang |
| 189 | Xin Tuan Hei Gu | Ninglang |
| 190 | Xiu Shui Gu | Jiangcheng |
| 191 | Yi Liang Xian | Fumin |
| 192 | Yuan Jiang Bai Gu | Mojiang |
| 193 | Yuan Jiang Zao Gu | Mojiang |

| ID | Variety | Origin |
|---|---|---|
| 194 | Yue Liang Gu | Yuanyang |
| 195 | Yun Xian Hong Mi | Honghe |
| 196 | Zao Diao | Fumin |
| 197 | Zao Er Kuai | Mojiang |
| 198 | Zao Gu | Gengma |
| 199 | Zi Gan Gu | Yongde |
| 200 | Zi Gu | Honghe |
| 201 | Zi Nuo | Lvchun |

*Yunnan Local Rice Germplasm Accessions Sharing Identical Names but Originating from Different Sources.

**Table S2.** Information of molecular markers of Waxy gene alleles.

| Markers | Primer Type | Primer Sequence |
|---|---|---|
| $Waxy^{gt}$-ARMS | Forward Inner Primer | GTTCATCAGGAAGAACATCTGCAATT |
| $Waxy^{gt}$-ARMS | Reverse Inner Primer | AAACAAAGAATTATAAACATA-TATGTATAC |
| $Waxy^{gt}$-ARMS | Forward Outer Primer | GTTCTTTGTCTATCTCAAGACACAAATAA |
| $Waxy^{gt}$-ARMS | Reverse Outer Primer | ATATATATGGATCTTGGCAAGTCAATTA |
| $Waxy^{ac}$-ARMS | Forward Inner Primer | AACAACCCATACTTCAAAGGAACTGA |
| $Waxy^{ac}$-ARMS | Reverse Inner Primer | GATCTTGAGATCAATTGTAACTCACCCG |
| $Waxy^{ac}$-ARMS | Forward Outer Primer | GATTCATAACTAATTCGTGTATTGATGC |
| $Waxy^{ac}$-ARMS | Reverse Outer Primer | AGTAACAAACCGGAAGGAATTAGTCT |

**Table S3.** Information of molecular markers of Indica-Japonica Classification.

| Markers | Forward Primer | Reverse Primer |
|---|---|---|
| R1M7 | ATTCCTGGTTCTACATTACTTA | CGCCTCACTAGAATATCGGA |
| R1M30 | AAGGGGCCCTAATTTATCTAG | TGTTTACTTTGTTCTTGGACTG |
| R1M37 | ATAGTTCGCCATCGTCAT | ACACGCCATAGCAAGGAA |
| R1M47 | AATAGAATTACTGATGAAACCTTA | GCCCGTTACCGCTTATGT |
| R2M10 | CCCAGTCTGCTGCCATCT | GAATGTATTTCAGTTCCAGTAAG |
| R2M24 | GGGCAACAACGGCTCTG | AGGGAATAAGGCGATACGG |
| R2M26 | GCAGCAAAGTGCGGAGTA | CAGGTGAATTGCCAATTT |
| R2M50 | CCTGAAGGAAATGATAGCAATAG | GTTTTGTATGCTCTTCACTTGTC |

| Markers | Forward Primer | Reverse Primer |
|---|---|---|
| R3M10 | CCGAGTACCATTGCTTTC | CTGCCATAGTTACTGCTCTGTT |
| R3M23 | TGCTTACAAGGGTCCAAT | GGAGGTGCCTACCAAGAG |
| R3M53 | ACACTGGCTACGGCAAAG | TTTGTTCGGGAATAATGATGC |
| R4M13 | TACACGGTAGACATCCAACA | ATGATTTAACCGTAGATTGG |
| R4M17 | AGTGCTCGGTTTGTTTTC | GTCAGATATAATTGATGGATGTA |
| R4M43 | CTTGAACCTGAGTGAGTGG | CGATGAAAATGATGTCTA |
| R5M13 | GAGAAAGAGTGGAAGGAG | AGTATCGTCAGGAGGGTC |
| R5M30 | CTCAATTTCACCCATCCC | CGCTCCGTCTCCAACCTC |
| R6M14 | AAATGTCCATGTGTTTGCTTC | CATGTGTGGAATGTGGTTG |
| R6M44 | TTAGGAATAAAGGCTGGATA | TTACCGTTAATAGGTGGAA |
| R7M7 | ACCTTCCCTCCCCTTTTGAT | AACTTGGTCTTCCTGTTTTATTG |
| R7M37 | CAGCCCTAAATCTAAATACCC | ACGTTGAGACAGGCGAGC |
| R8M23 | CCTATTCACTCTACCGACAT | GTTTAGTTCCCATTGCTTT |
| R8M33 | CGAAAGAGGAGAGGGGTAGT | CGAAAACGAGAAACAAATA |
| R8M46 | CAGCAGAGTCCAGAGAAGAT | GCATAAGATGGCGAGTGA |
| R9M10 | CTTTGGATTCAGGGGGA | AACTTGAAACGGAGGCAG |
| R9M42 | CTATAAGACCAAAACGAAAACT | GAAAACCATTGTGTCACTGTA |
| R10M17 | TGAACAATAAACCACAGAAGCA | CCCTTTATTCCCTCCTTTG |
| R10M30 | CCCTAAAAATAGAGCAACCT | ACCCATAATACTACCAATCAAC |
| R10M40 | GTCCCTAGGCCATCTCTTG | GCGAATAGGGGTGGACAG |
| R11M23 | AAGGTTGACAAGGACAGAAG | TCGCAGGAATGGATAAAA |
| R11M40 | AAGAAAAATATCTATTGAGGAGTG | GGAGGACCATAAATGACGG |
| R12M10 | ATCATTTCAGCCTGTGCC | AGCTTAATAGGGGGGACG |
| R12M27 | ATTTCATTGCCATCAGTT | GTAATCTTCTATCCGTTCA |
| R12M43 | CCGCCGAGAAGAAACAAAG | CCCAAGAACAGGATTACA |

**Table S4.** Criteria for Classification of Indica and Japonica Rice.

| Indica index | Japonica index | Indica/Japonica |
|---|---|---|
| ≥0.90 | ≤0.10 | typical indica |
| 0.75-0.89 | 0.11-0.25 | indica |
| 0.61-0.74 | 0.26-0.39 | indica-leaning |
| 0.40-0.60 | 0.40-0.60 | intermediate |

| | | |
|---|---|---|
| 0.26-0.39 | 0.61-0.74 | japonica-leaning |
| 0.11-0.25 | 0.75-0.89 | japonica |
| ≤0.10 | ≥0.90 | typical japonica |

**Table S5.** Details of Allelic Variations in the Waxy Gene across 201 Yunnan Local Rice Germplasm Accessions.

| ID | Variety | Waxy Geno-type | ID | Variety | Waxy Geno-type | ID | Variety | Waxy Geno-type |
|---|---|---|---|---|---|---|---|---|
| 1 | Ba Bao Gu | $Wx^a$ | 2 | Ba Qi | $Wx^a$ | 3 | Ba Yue Hong Gu | $Wx^a$ |
| 4 | Ba Yue Nuo | $Wx^b$ | 5 | Bai Gan Hong Gu(1)* | $Wx^b$ | 6 | Bai Gan Hong Gu(2)* | $Wx^a$ |
| 7 | Bai Gan Lu Feng | $Wx^a$ | 8 | Bai Gen Qi Tou | $Wx^a$ | 9 | Bai Gu | $Wx^a$ |
| 10 | Bai Gu Hong Mi | $Wx^a$ | 11 | Bai Hua Pi Nuo | $Wx^a$ | 12 | Bai Liang Gu | $Wx^a$ |
| 13 | Bai Mao Xiang Gu(1)* | $Wx^a$ | 14 | Bai Mao Xiang Gu(2)* | $Wx^a$ | 15 | Bai Mian Gu | $Wx^a$ |
| 16 | Bai Pi Hong Mi Ma Zha Gu | $Wx^a$ | 17 | Bai Xiang Gu | $Wx^b$ | 18 | Bai Xiang Nuo | $Wx^b$ |
| 19 | Bai Xiang Nuo Gu | $Wx^a$ | 20 | Ban He Yi Hao Gu | $Wx^a$ | 21 | Ban Jie Mang | $Wx^b$ |
| 22 | Bi Wu Sheng | $Wx^a$ | 23 | Bian Liang Gu | $Wx^a$ | 24 | Bie Wu Sheng | $Wx^a$ |
| 25 | Bo Li Nuo | $Wx^a$ | 26 | Cheng Du Ai | $Wx^a$ | 27 | Chi Bai Gu | $Wx^a$ |
| 28 | Chong Tui(1)* | $Wx^a$ | 29 | Chong Tui(2)* | $Wx^a$ | 30 | Chu Ya Gu | $Wx^a$ |
| 31 | Da Ai Ke | $Wx^a$ | 32 | Da Bai Gu(1)* | $Wx^a$ | 33 | Da Bai Gu(2)* | $Wx^a$ |
| 34 | Da Bai Gu(3)* | $Wx^a$ | 35 | Da Bai Nuo(1)* | $Wx^b$ | 36 | Da Bai Nuo(2)* | $Wx^a$ |
| 37 | Da Er Xiang | $Wx^b$ | 38 | Da Hei Gu | $Wx^a$ | 39 | Da Hei Ke Gu | $Wx^a$ |
| 40 | Da Hong Gu(1)* | $Wx^a$ | 41 | Da Hong Gu(2)* | $Wx^a$ | 42 | Da Hong Gu(3)* | $Wx^a$ |
| 43 | Da Hong Pi Nuo | $Wx^b$ | 44 | Da Hua Nuo | $Wx^a$ | 45 | Da Huang Pi Nuo | $Wx^a$ |

| ID | Variety | Waxy Genotype | ID | Variety | Waxy Genotype | ID | Variety | Waxy Genotype |
|---|---|---|---|---|---|---|---|---|
| 46 | Da Li Gu | $Wx^a$ | 47 | Da Long Gu | $Wx^a$ | 48 | Da Luo Ping | $Wx^a$ |
| 49 | Da Ma Zha Gu | $Wx^a$ | 50 | Da Qian Qian Gu | $Wx^a$ | 51 | Da Sha Ren Gu | $Wx^a$ |
| 52 | Da Wa Gu | $Wx^b$ | 53 | Da Zi Gan | $Wx^a$ | 54 | Ding Liang Gu | $Wx^a$ |
| 55 | Dong Gan Wu Ju | $Wx^a$ | 56 | Dong Li Hong Gu | $Wx^a$ | 57 | Duan Jiao Bai Gu | $Wx^a$ |
| 58 | Er Bai Diao | $Wx^a$ | 59 | Er Bai Gu | $Wx^a$ | 60 | Er Kuai Gu | $Wx^a$ |
| 61 | Gan Zha Gu | $Wx^a$ | 62 | Gao Liang Nuo | $Wx^b$ | 63 | Gao San Sui | $Wx^a$ |
| 64 | Gao Tai Ba Hao | $Wx^a$ | 65 | Guang Nan Gu | $Wx^a$ | 66 | Guangnan Yi Hao | $Wx^a$ |
| 67 | Hai Gu | $Wx^a$ | 68 | Han Luo Ping | $Wx^a$ | 69 | Hao An Wang | $Wx^a$ |
| 70 | Hao An Wang Mie Duan | $Wx^a$ | 71 | Hao Ba Wan | $Wx^a$ | 72 | Hao Bu Ka | $Wx^a$ |
| 73 | Hao Dong Nan | $Wx^a$ | 74 | Hao Gan | $Wx^a$ | 75 | Hao Ge Lao | $Wx^a$ |
| 76 | Hao Hao Nen | $Wx^b$ | 77 | Hao Hua Xiang | $Wx^a$ | 78 | Hao Jiang Duo | $Wx^a$ |
| 79 | Hao Ma Liang | $Wx^a$ | 80 | Hao Men Lia | $Wx^a$ | 81 | Hao Mie Han | $Wx^a$ |
| 82 | Hao Mu Lei | $Wx^a$ | 83 | Hao Mu Xi(1)* | $Wx^a$ | 84 | Hao Mu Xi(2)* | $Wx^a$ |
| 85 | Hao Yu Bang Nan | $Wx^a$ | 86 | Hao Zao Hao | $Wx^a$ | 87 | Hei Zi Gan | $Wx^a$ |
| 88 | Hei Zi Mi | $Wx^b$ | 89 | Hong Gan Ma Zha Gu | $Wx^a$ | 90 | Hong Gu(1)* | $Wx^a$ |
| 91 | Hong Gu(2)* | $Wx^a$ | 92 | Hong Gu(3)* | $Wx^{in}$ | 93 | Hong Han Nuo | $Wx^a$ |
| 94 | Hong He Hong Mi | $Wx^a$ | 95 | Hong Hua Gu | $Wx^a$ | 96 | Hong Hua Mi | $Wx^a$ |
| 97 | Hong Jiao Bai | $Wx^a$ | 98 | Hong Mi | $Wx^a$ | 99 | Hong Mi Ma Zhan | $Wx^a$ |
| 100 | Hong Pi Xiang | $Wx^a$ | 101 | Hong Xiang Gu(1)* | $Wx^a$ | 102 | Hong Xiang Gu(2)* | $Wx^a$ |
| 103 | Hong Yun Dang | $Wx^a$ | 104 | Hong Zao Gu | $Wx^a$ | 105 | Hua Er Zao | $Wx^a$ |

| ID | Variety | Waxy Genotype | ID | Variety | Waxy Genotype | ID | Variety | Waxy Genotype |
|---|---|---|---|---|---|---|---|---|
| 106 | Hua Gu San Bai Bang | $Wx^{mw}$ | 107 | Hua Pi Hong Gu | $Wx^{a}$ | 108 | Huang Ban Suo | $Wx^{a}$ |
| 109 | Huang Hui Xiang | $Wx^{a}$ | 110 | Huang Pi Ruan Mi | $Wx^{a}$ | 111 | Huang Pi Xiang | $Wx^{a}$ |
| 112 | Huang Xiang Nuo | $Wx^{b}$ | 113 | Huang Xin Nuo | $Wx^{a}$ | 114 | Ji You Nuo | $Wx^{a}$ |
| 115 | Jia Xiang | $Wx^{a}$ | 116 | Jian Tang Zao | $Wx^{a}$ | 117 | Jie Gu Nuo(1)* | $Wx^{a}$ |
| 118 | Jie Gu Nuo(2)* | $Wx^{b}$ | 119 | Jie Gu Nuo(3)* | $Wx^{b}$ | 120 | Jin Bao Yin | $Wx^{b}$ |
| 121 | Jiu Yue Gu | $Wx^{a}$ | 122 | Jiu Yue Nuo | $Wx^{a}$ | 123 | Kun Ming Xiao Bai Gu | $Wx^{a}$ |
| 124 | Lan Pi Gu | $Wx^{a}$ | 125 | Lao Gong Gu | $Wx^{a}$ | 126 | Lao Gua Gu | $Wx^{a}$ |
| 127 | Lao Xiang Gu | $Wx^{a}$ | 128 | Lao Yang Gu | $Wx^{a}$ | 129 | Leng Shui Bai Gu | $Wx^{a}$ |
| 130 | Long Gu | $Wx^{a}$ | 131 | Lu Shui Nuo | $Wx^{a}$ | 132 | Luan Jiao Bai Gu | $Wx^{a}$ |
| 133 | Luan Jiao Diao | $Wx^{a}$ | 134 | Lue Ha | $Wx^{a}$ | 135 | Luo Ping Gu | $Wx^{a}$ |
| 136 | Ma Deng Hong Gu | $Wx^{a}$ | 137 | Ma Ti Gu | $Wx^{a}$ | 138 | Ma Xian Gu(1)* | $Wx^{a}$ |
| 139 | Ma Xian Gu(2)* | $Wx^{b}$ | 140 | Ma Zha Gu(1)* | $Wx^{b}$ | 141 | Ma Zha Gu(2)* | $Wx^{a}$ |
| 142 | Ma Zha Gu(3)* | $Wx^{a}$ | 143 | Ma Zha Gu(4)* | $Wx^{a}$ | 144 | Ma Zha Gu(5)* | $Wx^{a}$ |
| 145 | Ma Zha Nuo | $Wx^{a}$ | 146 | Mang Shi Gu | $Wx^{a}$ | 147 | Mang Zhong Gu | $Wx^{a}$ |
| 148 | Mao Ke Hong Nuo | $Wx^{a}$ | 149 | Mao Xiang Gu | $Wx^{a}$ | 150 | Mao Xiang Nuo | $Wx^{a}$ |
| 151 | Meng Ka Zha | $Wx^{a}$ | 152 | Meng Lu Gu | $Wx^{a}$ | 153 | Meng Wang Gu(1)* | $Wx^{b}$ |
| 154 | Meng Wang Gu(2)* | $Wx^{mw}$ | 155 | Meng Xian Da Bai Gu | $Wx^{a}$ | 156 | Mo Wang Gu | $Wx^{a}$ |
| 157 | Niao Ju Qi Tou | $Wx^{a}$ | 158 | Niu Yang Gu | $Wx^{a}$ | 159 | Ou Yang Gu | $Wx^{a}$ |

| ID | Variety | Waxy Genotype | ID | Variety | Waxy Genotype | ID | Variety | Waxy Genotype |
|---|---|---|---|---|---|---|---|---|
| 160 | Qi Tou Gu | $Wx^a$ | 161 | Qi Yue Nuo | $Wx^a$ | 162 | Qing Ming Li Hong Gu | $Wx^a$ |
| 163 | Shan Dao Hong Gu | $Wx^a$ | 164 | Ta Pi Gu | $Wx^a$ | 165 | Tu Po Huo Lue | $Wx^a$ |
| 166 | Tuan Ke Li | $Wx^a$ | 167 | Wu Bai Li | $Wx^a$ | 168 | Wu Ju Gu | $Wx^a$ |
| 169 | Xi Bai Gu(1)* | $Wx^a$ | 170 | Xi Bai Gu(2)* | $Wx^a$ | 171 | Xi Gan Qi Tou | $Wx^a$ |
| 172 | Xi Luo Ping | $Wx^a$ | 173 | Xi Ma Xian Gu | $Wx^a$ | 174 | Xi Ma Zha Gu | $Wx^b$ |
| 175 | Xiang Diao | $Wx^a$ | 176 | Xiang Gu(1)* | $Wx^a$ | 177 | Xiang Gu(2)* | $Wx^a$ |
| 178 | Xiang Gu(3)* | $Wx^a$ | 179 | Xiang Nuo | $Wx^a$ | 180 | Xiao Bai Gu | $Wx^a$ |
| 181 | Xiao Bai Nuo | $Wx^a$ | 182 | Xiao Hong Gu(1)* | $Wx^a$ | 183 | Xiao Hong Gu(2)* | $Wx^a$ |
| 184 | Xiao Hong Gu(3)* | $Wx^a$ | 185 | Xiao Luo Ping | $Wx^a$ | 186 | Xiao Ma Zha Gu | $Wx^{in}$ |
| 187 | Xiao Wu Ju Gu | $Wx^a$ | 188 | Xiao Zi Gan | $Wx^a$ | 189 | Xin Tuan Hei Gu | $Wx^{in}$ |
| 190 | Xiu Shui Gu | $Wx^a$ | 191 | Yi Liang Xian | $Wx^a$ | 192 | Yuan Jiang Bai Gu | $Wx^a$ |
| 193 | Yuan Jiang Zao Gu | $Wx^a$ | 194 | Yue Liang Gu | $Wx^a$ | 195 | Yun Xian Hong Mi | $Wx^a$ |
| 196 | Zao Diao | $Wx^a$ | 197 | Zao Er Kuai | $Wx^a$ | 198 | Zao Gu | $Wx^a$ |
| 199 | Zi Gan Gu | $Wx^a$ | 200 | Zi Gu | $Wx^{in}$ | 201 | Zi Nuo | $Wx^a$ |

*Yunnan Local Rice Germplasm Accessions Sharing Identical Names but Originating from Different Sources.

**Table S6.** Amylose Content Associated with Different Waxy Genotypes in 201 Yunnan Local Rice Germplasm Accessions.

| ID | Variety | Waxy Genotype | Indica Index | Indica-Japonica Attribute |
|---|---|---|---|---|
| 1 | Ba Bao Gu | Wxa | 0.89 | Indica Rice |
| 2 | Ba Qi | Wxa | 1 | Typical Indica |
| 3 | Ba Yue Hong Gu | Wxa | 0.87 | Indica Rice |
| 4 | Ba Yue Nuo | Wxb | 0.33 | Leaning Japonica |
| 5 | Bai Gan Hong Gu(1)* | Wxb | 0.07 | Typical Japonica |
| 6 | Bai Gan Hong Gu(2)* | Wxa | 1 | Typical Indica |
| 7 | Bai Gan Lu Feng | Wxa | 0.97 | Typical Indica |
| 8 | Bai Gen Qi Tou | Wxa | 0.96 | Typical Indica |
| 9 | Bai Gu | Wxa | 1 | Typical Indica |

| ID | Variety | Waxy Genotype | Indica Index | Indica-Japonica Attribute |
|---|---|---|---|---|
| 10 | Bai Gu Hong Mi | Wxa | 0.88 | Indica Rice |
| 11 | Bai Hua Pi Nuo | Wxa | 0.93 | Typical Indica |
| 12 | Bai Liang Gu | Wxa | 1 | Typical Indica |
| 13 | Bai Mao Xiang Gu(1)* | Wxa | 0.68 | Leaning Indica |
| 14 | Bai Mao Xiang Gu(2)* | Wxa | 0.97 | Typical Indica |
| 15 | Bai Mian Gu | Wxa | 0.91 | Typical Indica |
| 16 | Bai Pi Hong Mi Ma Zha Gu | Wxa | 0.97 | Typical Indica |
| 17 | Bai Xiang Gu | Wxb | 0.16 | Japonica Rice |
| 18 | Bai Xiang Nuo | Wxb | 0.15 | Japonica Rice |
| 19 | Bai Xiang Nuo Gu | Wxa | 0.91 | Typical Indica |
| 20 | Ban He Yi Hao Gu | Wxa | 0.98 | Typical Indica |
| 21 | Ban Jie Mang | Wxb | 0.08 | Typical Japonica |
| 22 | Bi Wu Sheng | Wxa | 0.94 | Typical Indica |
| 23 | Bian Liang Gu | Wxa | 0.7 | Leaning Indica |
| 24 | Bie Wu Sheng | Wxa | 0.85 | Indica Rice |
| 25 | Bo Li Nuo | Wxa | 0.87 | Indica Rice |
| 26 | Cheng Du Ai | Wxa | 1 | Typical Indica |
| 27 | Chi Bai Gu | Wxa | 0.97 | Typical Indica |
| 28 | Chong Tui(1)* | Wxa | 0.77 | Indica Rice |
| 29 | Chong Tui(2)* | Wxa | 0.81 | Indica Rice |
| 30 | Chu Ya Gu | Wxa | 0.97 | Typical Indica |
| 31 | Da Ai Ke | Wxa | 0.84 | Indica Rice |
| 32 | Da Bai Gu(1)* | Wxa | 0.91 | Typical Indica |
| 33 | Da Bai Gu(2)* | Wxa | 1 | Typical Indica |
| 34 | Da Bai Gu(3)* | Wxa | 0.84 | Indica Rice |
| 35 | Da Bai Nuo(1)* | Wxb | 0.21 | Japonica Rice |
| 36 | Da Bai Nuo(2)* | Wxa | 0.84 | Indica Rice |
| 37 | Da Er Xiang | Wxb | 0.11 | Japonica Rice |
| 38 | Da Hei Gu | Wxa | 0.91 | Typical Indica |
| 39 | Da Hei Ke Gu | Wxa | 0.87 | Indica Rice |
| 40 | Da Hong Gu(1)* | Wxa | 0.91 | Typical Indica |
| 41 | Da Hong Gu(2)* | Wxa | 0.97 | Typical Indica |
| 42 | Da Hong Gu(3)* | Wxa | 0.88 | Indica Rice |
| 43 | Da Hong Pi Nuo | Wxb | 0.25 | Leaning Japonica |
| 44 | Da Hua Nuo | Wxa | 0.9 | Typical Indica |
| 45 | Da Huang Pi Nuo | Wxa | 0.94 | Typical Indica |
| 46 | Da Li Gu | Wxa | 0.83 | Indica Rice |
| 47 | Da Long Gu | Wxa | 0.92 | Typical Indica |
| 48 | Da Luo Ping | Wxa | 0.88 | Indica Rice |
| 49 | Da Ma Zha Gu | Wxa | 0.97 | Typical Indica |
| 50 | Da Qian Qian Gu | Wxa | 0.87 | Indica Rice |
| 51 | Da Sha Ren Gu | Wxa | 1 | Typical Indica |
| 52 | Da Wa Gu | Wxb | 0.97 | Typical Indica |

| ID | Variety | Waxy Genotype | Indica Index | Indica-Japonica Attribute |
|---|---|---|---|---|
| 53 | Da Zi Gan | Wxa | 0.93 | Typical Indica |
| 54 | Ding Liang Gu | Wxa | 0.97 | Typical Indica |
| 55 | Dong Gan Wu Ju | Wxa | 0.91 | Typical Indica |
| 56 | Dong Li Hong Gu | Wxa | 0.88 | Indica Rice |
| 57 | Duan Jiao Bai Gu | Wxa | 0.94 | Typical Indica |
| 58 | Er Bai Diao | Wxa | 0.82 | Indica Rice |
| 59 | Er Bai Gu | Wxa | 0.93 | Typical Indica |
| 60 | Er Kuai Gu | Wxa | 0.62 | Leaning Indica |
| 61 | Gan Zha Gu | Wxa | 0.93 | Typical Indica |
| 62 | Gao Liang Nuo | Wxb | 0.94 | Typical Indica |
| 63 | Gao San Sui | Wxa | 0.97 | Typical Indica |
| 64 | Gao Tai Ba Hao | Wxa | 0.92 | Typical Indica |
| 65 | Guang Nan Gu | Wxa | 0.86 | Indica Rice |
| 66 | Guangnan Yi Hao | Wxa | 0.88 | Indica Rice |
| 67 | Hai Gu | Wxa | 0.97 | Typical Indica |
| 68 | Han Luo Ping | Wxa | 0.92 | Typical Indica |
| 69 | Hao An Wang | Wxa | 0.91 | Typical Indica |
| 70 | Hao An Wang Mie Duan | Wxa | 0.91 | Typical Indica |
| 71 | Hao Ba Wan | Wxa | 0.94 | Typical Indica |
| 72 | Hao Bu Ka | Wxa | 0.86 | Indica Rice |
| 73 | Hao Dong Nan | Wxa | 1 | Typical Indica |
| 74 | Hao Gan | Wxa | 0.83 | Indica Rice |
| 75 | Hao Ge Lao | Wxa | 0.64 | Leaning Indica |
| 76 | Hao Hao Nen | Wxb | 0.92 | Typical Indica |
| 77 | Hao Hua Xiang | Wxa | 0.86 | Indica Rice |
| 78 | Hao Jiang Duo | Wxa | 0.98 | Typical Indica |
| 79 | Hao Ma Liang | Wxa | 0.94 | Typical Indica |
| 80 | Hao Men Lia | Wxa | 0.86 | Indica Rice |
| 81 | Hao Mie Han | Wxa | 0.79 | Indica Rice |
| 82 | Hao Mu Lei | Wxa | 0.91 | Typical Indica |
| 83 | Hao Mu Xi(1)* | Wxa | 0.91 | Typical Indica |
| 84 | Hao Mu Xi(2)* | Wxa | 0.92 | Typical Indica |
| 85 | Hao Yu Bang Nan | Wxa | 0.9 | Typical Indica |
| 86 | Hao Zao Hao | Wxa | 0.55 | Intermediate Type |
| 87 | Hei Zi Gan | Wxa | 0.93 | Typical Indica |
| 88 | Hei Zi Mi | Wxb | 0.16 | Japonica Rice |
| 89 | Hong Gan Ma Zha Gu | Wxa | 0.9 | Typical Indica |
| 90 | Hong Gu(1)* | Wxa | 0.91 | Typical Indica |
| 91 | Hong Gu(2)* | Wxa | 0.89 | Indica Rice |
| 92 | Hong Gu(3)* | Wxin | 0.92 | Typical Indica |
| 93 | Hong Han Nuo | Wxa | 0.89 | Indica Rice |
| 94 | Hong He Hong Mi | Wxa | 0.95 | Typical Indica |
| 95 | Hong Hua Gu | Wxa | 1 | Typical Indica |

| ID | Variety | Waxy Genotype | Indica Index | Indica-Japonica Attribute |
|---|---|---|---|---|
| 96 | Hong Hua Mi | Wxa | 1 | Typical Indica |
| 97 | Hong Jiao Bai | Wxa | 0.94 | Typical Indica |
| 98 | Hong Mi | Wxa | 0.83 | Indica Rice |
| 99 | Hong Mi Ma Zhan | Wxa | 0.93 | Typical Indica |
| 100 | Hong Pi Xiang | Wxa | 0.94 | Typical Indica |
| 101 | Hong Xiang Gu(1)* | Wxa | 0.88 | Indica Rice |
| 102 | Hong Xiang Gu(2)* | Wxa | 0.9 | Typical Indica |
| 103 | Hong Yun Dang | Wxa | 0.91 | Typical Indica |
| 104 | Hong Zao Gu | Wxa | 0.97 | Typical Indica |
| 105 | Hua Er Zao | Wxa | 0.7 | Leaning Indica |
| 106 | Hua Gu San Bai Bang | Wxmw | 0.13 | Japonica Rice |
| 107 | Hua Pi Hong Gu | Wxa | 0.94 | Typical Indica |
| 108 | Huang Ban Suo | Wxa | 0.95 | Typical Indica |
| 109 | Huang Hui Xiang | Wxa | 0.91 | Typical Indica |
| 110 | Huang Pi Ruan Mi | Wxa | 0.89 | Indica Rice |
| 111 | Huang Pi Xiang | Wxa | 0.9 | Typical Indica |
| 112 | Huang Xiang Nuo | Wxb | 0.06 | Typical Japonica |
| 113 | Huang Xin Nuo | Wxa | 0.67 | Leaning Indica |
| 114 | Ji You Nuo | Wxa | 0.95 | Typical Indica |
| 115 | Jia Xiang | Wxa | 0.94 | Typical Indica |
| 116 | Jian Tang Zao | Wxa | 0.93 | Typical Indica |
| 117 | Jie Gu Nuo(1)* | Wxa | 0.81 | Indica Rice |
| 118 | Jie Gu Nuo(2)* | Wxb | 0.18 | Japonica Rice |
| 119 | Jie Gu Nuo(3)* | Wxb | 0.85 | Indica Rice |
| 120 | Jin Bao Yin | Wxb | 0.05 | Typical Japonica |
| 121 | Jiu Yue Gu | Wxa | 0.97 | Typical Indica |
| 122 | Jiu Yue Nuo | Wxa | 0.95 | Typical Indica |
| 123 | Kun Ming Xiao Bai Gu | Wxa | 0.84 | Indica Rice |
| 124 | Lan Pi Gu | Wxa | 0.91 | Typical Indica |
| 125 | Lao Gong Gu | Wxa | 0.98 | Typical Indica |
| 126 | Lao Gua Gu | Wxa | 0.94 | Typical Indica |
| 127 | Lao Xiang Gu | Wxa | 0.94 | Typical Indica |
| 128 | Lao Yang Gu | Wxa | 0.95 | Typical Indica |
| 129 | Leng Shui Bai Gu | Wxa | 0.95 | Typical Indica |
| 130 | Long Gu | Wxa | 1 | Typical Indica |
| 131 | Lu Shui Nuo | Wxa | 0.89 | Indica Rice |
| 132 | Luan Jiao Bai Gu | Wxa | 1 | Typical Indica |
| 133 | Luan Jiao Diao | Wxa | 0.85 | Indica Rice |
| 134 | Lue Ha | Wxa | 0.97 | Typical Indica |
| 135 | Luo Ping Gu | Wxa | 0.91 | Typical Indica |
| 136 | Ma Deng Hong Gu | Wxa | 0.91 | Typical Indica |
| 137 | Ma Ti Gu | Wxa | 0.94 | Typical Indica |
| 138 | Ma Xian Gu(1)* | Wxa | 1 | Typical Indica |

| ID | Variety | Waxy Genotype | Indica Index | Indica-Japonica Attribute |
| --- | --- | --- | --- | --- |
| 139 | Ma Xian Gu(2)* | Wxb | 0.13 | Japonica Rice |
| 140 | Ma Zha Gu(1)* | Wxb | 0.94 | Typical Indica |
| 141 | Ma Zha Gu(2)* | Wxa | 0.81 | Indica Rice |
| 142 | Ma Zha Gu(3)* | Wxa | 0.91 | Typical Indica |
| 143 | Ma Zha Gu(4)* | Wxa | 0.87 | Indica Rice |
| 144 | Ma Zha Gu(5)* | Wxa | 1 | Typical Indica |
| 145 | Ma Zha Nuo | Wxa | 0.94 | Typical Indica |
| 146 | Mang Shi Gu | Wxa | 0.9 | Typical Indica |
| 147 | Mang Zhong Gu | Wxa | 0.84 | Indica Rice |
| 148 | Mao Ke Hong Nuo | Wxa | 0.94 | Typical Indica |
| 149 | Mao Xiang Gu | Wxa | 0.82 | Indica Rice |
| 150 | Mao Xiang Nuo | Wxa | 0.42 | Intermediate Type |
| 151 | Meng Ka Zha | Wxa | 0.76 | Indica Rice |
| 152 | Meng Lu Gu | Wxa | 0.94 | Typical Indica |
| 153 | Meng Wang Gu(1)* | Wxb | 0.03 | Typical Japonica |
| 154 | Meng Wang Gu(2)* | Wxmw | 0.23 | Japonica Rice |
| 155 | Meng Xian Da Bai Gu | Wxa | 0.97 | Typical Indica |
| 156 | Mo Wang Gu | Wxa | 0.87 | Indica Rice |
| 157 | Niao Ju Qi Tou | Wxa | 0.97 | Typical Indica |
| 158 | Niu Yang Gu | Wxa | 0.83 | Indica Rice |
| 159 | Ou Yang Gu | Wxa | 0.91 | Typical Indica |
| 160 | Qi Tou Gu | Wxa | 0.84 | Indica Rice |
| 161 | Qi Yue Nuo | Wxa | 0.94 | Typical Indica |
| 162 | Qing Ming Li Hong Gu | Wxa | 0.88 | Indica Rice |
| 163 | Shan Dao Hong Gu | Wxa | 0.9 | Typical Indica |
| 164 | Ta Pi Gu | Wxa | 0.98 | Typical Indica |
| 165 | Tu Po Huo Lue | Wxa | 0.94 | Typical Indica |
| 166 | Tuan Ke Li | Wxa | 0.94 | Typical Indica |
| 167 | Wu Bai Li | Wxa | 0.85 | Indica Rice |
| 168 | Wu Ju Gu | Wxa | 0.94 | Typical Indica |
| 169 | Xi Bai Gu(1)* | Wxa | 0.91 | Typical Indica |
| 170 | Xi Bai Gu(2)* | Wxa | 0.9 | Typical Indica |
| 171 | Xi Gan Qi Tou | Wxa | 0.83 | Indica Rice |
| 172 | Xi Luo Ping | Wxa | 0.94 | Typical Indica |
| 173 | Xi Ma Xian Gu | Wxa | 0.86 | Indica Rice |
| 174 | Xi Ma Zha Gu | Wxb | 0.09 | Typical Japonica |
| 175 | Xiang Diao | Wxa | 0.92 | Typical Indica |
| 176 | Xiang Gu(1)* | Wxa | 0.92 | Typical Indica |
| 177 | Xiang Gu(2)* | Wxa | 1 | Typical Indica |
| 178 | Xiang Gu(3)* | Wxa | 0.91 | Typical Indica |
| 179 | Xiang Nuo | Wxa | 0.91 | Typical Indica |
| 180 | Xiao Bai Gu | Wxa | 0.91 | Typical Indica |
| 181 | Xiao Bai Nuo | Wxa | 0.85 | Indica Rice |

| **ID** | **Variety** | **Waxy Genotype** | **Indica Index** | **Indica-Japonica Attribute** |
|---|---|---|---|---|
| 182 | Xiao Hong Gu(1)* | Wxa | 0.82 | Indica Rice |
| 183 | Xiao Hong Gu(2)* | Wxa | 0.97 | Typical Indica |
| 184 | Xiao Hong Gu(3)* | Wxa | 0.97 | Typical Indica |
| 185 | Xiao Luo Ping | Wxa | 0.94 | Typical Indica |
| 186 | Xiao Ma Zha Gu | Wxin | 0.86 | Indica Rice |
| 187 | Xiao Wu Ju Gu | Wxa | 0.89 | Typical Indica |
| 188 | Xiao Zi Gan | Wxa | 0.88 | Indica Rice |
| 189 | Xin Tuan Hei Gu | Wxin | 0.06 | Typical Japonica |
| 190 | Xiu Shui Gu | Wxa | 1 | Typical Indica |
| 191 | Yi Liang Xian | Wxa | 0.97 | Typical Indica |
| 192 | Yuan Jiang Bai Gu | Wxa | 0.9 | Typical Indica |
| 193 | Yuan Jiang Zao Gu | Wxa | 0.94 | Typical Indica |
| 194 | Yue Liang Gu | Wxa | 0.94 | Typical Indica |
| 195 | Yun Xian Hong Mi | Wxa | 0.96 | Typical Indica |
| 196 | Zao Diao | Wxa | 0.85 | Indica Rice |
| 197 | Zao Er Kuai | Wxa | 0.8 | Indica Rice |
| 198 | Zao Gu | Wxa | 0.97 | Typical Indica |
| 199 | Zi Gan Gu | Wxa | 0.97 | Typical Indica |
| 200 | Zi Gu | Wxin | 0.07 | Typical Japonica |
| 201 | Zi Nuo | Wxa | 0.88 | Indica Rice |

*Yunnan Local Rice Germplasm Accessions Sharing Identical Names but Originating from Different Sources.

**Table S7.** Indica-Japonica Attributes Associated with Different Waxy Genotypes in 201 Yunnan Local Rice Germplasm Accessions.

| ID | Variety | Waxy Genotype | Amylose Content |
|---|---|---|---|
| 1 | Ba Bao Gu | Wxa | 0.2723 |
| 2 | Ba Qi | Wxa | 0.2603 |
| 3 | Ba Yue Hong Gu | Wxa | 0.2231 |
| 4 | Ba Yue Nuo | Wxb | 0.0916 |
| 5 | Bai Gan Hong Gu(1)* | Wxb | 0.1031 |
| 6 | Bai Gan Hong Gu(2)* | Wxa | 0.2788 |
| 7 | Bai Gan Lu Feng | Wxa | 0.2578 |
| 8 | Bai Gen Qi Tou | Wxa | 0.2477 |
| 9 | Bai Gu | Wxa | 0.2286 |
| 10 | Bai Gu Hong Mi | Wxa | 0.2156 |
| 11 | Bai Hua Pi Nuo | Wxa | 0.1915 |
| 12 | Bai Liang Gu | Wxa | 0.2527 |
| 13 | Bai Mao Xiang Gu(1)* | Wxa | 0.1769 |
| 14 | Bai Mao Xiang Gu(2)* | Wxa | 0.1543 |
| 15 | Bai Mian Gu | Wxa | 0.2367 |
| 16 | Bai Pi Hong Mi Ma Zha Gu | Wxa | 0.2397 |
| 17 | Bai Xiang Gu | Wxb | 0.1433 |
| 18 | Bai Xiang Nuo | Wxb | 0.1323 |

| | | | |
|---|---|---|---|
| 19 | Bai Xiang Nuo Gu | Wxa | 0.2166 |
| 20 | Ban He Yi Hao Gu | Wxa | 0.2342 |
| 21 | Ban Jie Mang | Wxb | 0.1393 |
| 22 | Bi Wu Sheng | Wxa | 0.2532 |
| 23 | Bian Liang Gu | Wxa | 0.2407 |
| 24 | Bie Wu Sheng | Wxa | 0.2347 |
| 25 | Bo Li Nuo | Wxa | 0.2447 |
| 26 | Cheng Du Ai | Wxa | 0.2497 |
| 27 | Chi Bai Gu | Wxa | 0.2984 |
| 28 | Chong Tui(1)* | Wxa | 0.191 |
| 29 | Chong Tui(2)* | Wxa | 0.2342 |
| 30 | Chu Ya Gu | Wxa | 0.2578 |
| 31 | Da Ai Ke | Wxa | 0.2497 |
| 32 | Da Bai Gu(1)* | Wxa | 0.2477 |
| 33 | Da Bai Gu(2)* | Wxa | 0.2808 |
| 34 | Da Bai Gu(3)* | Wxa | 0.2542 |
| 35 | Da Bai Nuo(1)* | Wxb | 0.1092 |
| 36 | Da Bai Nuo(2)* | Wxa | 0.2527 |
| 37 | Da Er Xiang | Wxb | 0.0946 |
| 38 | Da Hei Gu | Wxa | 0.2688 |
| 39 | Da Hei Ke Gu | Wxa | 0.2567 |
| 40 | Da Hong Gu(1)* | Wxa | 0.2588 |
| 41 | Da Hong Gu(2)* | Wxa | 0.2593 |
| 42 | Da Hong Gu(3)* | Wxa | 0.2673 |
| 43 | Da Hong Pi Nuo | Wxb | 0.1614 |
| 44 | Da Hua Nuo | Wxa | 0.1985 |
| 45 | Da Huang Pi Nuo | Wxa | 0.2417 |
| 46 | Da Li Gu | Wxa | 0.2417 |
| 47 | Da Long Gu | Wxa | 0.2588 |
| 48 | Da Luo Ping | Wxa | 0.2397 |
| 49 | Da Ma Zha Gu | Wxa | 0.2231 |
| 50 | Da Qian Qian Gu | Wxa | 0.0876 |
| 51 | Da Sha Ren Gu | Wxa | 0.2693 |
| 52 | Da Wa Gu | Wxb | 0.2146 |
| 53 | Da Zi Gan | Wxa | 0.2076 |
| 54 | Ding Liang Gu | Wxa | 0.2748 |
| 55 | Dong Gan Wu Ju | Wxa | 0.2437 |
| 56 | Dong Li Hong Gu | Wxa | 0.2497 |
| 57 | Duan Jiao Bai Gu | Wxa | 0.2688 |
| 58 | Er Bai Diao | Wxa | 0.2191 |
| 59 | Er Bai Gu | Wxa | 0.2447 |
| 60 | Er Kuai Gu | Wxa | 0.2447 |
| 61 | Gan Zha Gu | Wxa | 0.2628 |
| 62 | Gao Liang Nuo | Wxb | 0.2482 |

| | | | |
|---|---|---|---|
| 63 | Gao San Sui | Wxa | 0.2954 |
| 64 | Gao Tai Ba Hao | Wxa | 0.1789 |
| 65 | Guang Nan Gu | Wxa | 0.2271 |
| 66 | Guangnan Yi Hao | Wxa | 0.195 |
| 67 | Hai Gu | Wxa | 0.2839 |
| 68 | Han Luo Ping | Wxa | 0.2673 |
| 69 | Hao An Wang | Wxa | 0.0826 |
| 70 | Hao An Wang Mie Duan | Wxa | 0.1051 |
| 71 | Hao Ba Wan | Wxa | 0.1036 |
| 72 | Hao Bu Ka | Wxa | 0.2583 |
| 73 | Hao Dong Nan | Wxa | 0.2126 |
| 74 | Hao Gan | Wxa | 0.1373 |
| 75 | Hao Ge Lao | Wxa | 0.203 |
| 76 | Hao Hao Nen | Wxb | 0.0833 |
| 77 | Hao Hua Xiang | Wxa | 0.1493 |
| 78 | Hao Jiang Duo | Wxa | 0.0841 |
| 79 | Hao Ma Liang | Wxa | 0.2522 |
| 80 | Hao Men Lia | Wxa | 0.1398 |
| 81 | Hao Mie Han | Wxa | 0.1142 |
| 82 | Hao Mu Lei | Wxa | 0.0906 |
| 83 | Hao Mu Xi(1)* | Wxa | 0.1026 |
| 84 | Hao Mu Xi(2)* | Wxa | 0.1177 |
| 85 | Hao Yu Bang Nan | Wxa | 0.1955 |
| 86 | Hao Zao Hao | Wxa | 0.1473 |
| 87 | Hei Zi Gan | Wxa | 0.2497 |
| 88 | Hei Zi Mi | Wxb | 0.1443 |
| 89 | Hong Gan Ma Zha Gu | Wxa | 0.183 |
| 90 | Hong Gu(1)* | Wxa | 0.198 |
| 91 | Hong Gu(2)* | Wxa | 0.2567 |
| 92 | Hong Gu(3)* | Wxin | 0.2839 |
| 93 | Hong Han Nuo | Wxa | 0.2507 |
| 94 | Hong He Hong Mi | Wxa | 0.1323 |
| 95 | Hong Hua Gu | Wxa | 0.2532 |
| 96 | Hong Hua Mi | Wxa | 0.2517 |
| 97 | Hong Jiao Bai | Wxa | 0.2512 |
| 98 | Hong Mi | Wxa | 0.2256 |
| 99 | Hong Mi Ma Zhan | Wxa | 0.2417 |
| 100 | Hong Pi Xiang | Wxa | 0.2176 |
| 101 | Hong Xiang Gu(1)* | Wxa | 0.1639 |
| 102 | Hong Xiang Gu(2)* | Wxa | 0.2713 |
| 103 | Hong Yun Dang | Wxa | 0.1965 |
| 104 | Hong Zao Gu | Wxa | 0.2643 |
| 105 | Hua Er Zao | Wxa | 0.2347 |
| 106 | Hua Gu San Bai Bang | Wxmw | 0.19 |

| | | | |
|---|---|---|---|
| 107 | Hua Pi Hong Gu | Wxa | 0.203 |
| 108 | Huang Ban Suo | Wxa | 0.0931 |
| 109 | Huang Hui Xiang | Wxa | 0.249 |
| 110 | Huang Pi Ruan Mi | Wxa | 0.2623 |
| 111 | Huang Pi Xiang | Wxa | 0.2382 |
| 112 | Huang Xiang Nuo | Wxb | 0.1016 |
| 113 | Huang Xin Nuo | Wxa | 0.1026 |
| 114 | Ji You Nuo | Wxa | 0.1714 |
| 115 | Jia Xiang | Wxa | 0.184 |
| 116 | Jian Tang Zao | Wxa | 0.185 |
| 117 | Jie Gu Nuo(1)* | Wxa | 0.0881 |
| 118 | Jie Gu Nuo(2)* | Wxb | 0.0956 |
| 119 | Jie Gu Nuo(3)* | Wxb | 0.1423 |
| 120 | Jin Bao Yin | Wxb | 0.1036 |
| 121 | Jiu Yue Gu | Wxa | 0.2487 |
| 122 | Jiu Yue Nuo | Wxa | 0.2663 |
| 123 | Kun Ming Xiao Bai Gu | Wxa | 0.2502 |
| 124 | Lan Pi Gu | Wxa | 0.2452 |
| 125 | Lao Gong Gu | Wxa | 0.2517 |
| 126 | Lao Gua Gu | Wxa | 0.2266 |
| 127 | Lao Xiang Gu | Wxa | 0.2718 |
| 128 | Lao Yang Gu | Wxa | 0.2668 |
| 129 | Leng Shui Bai Gu | Wxa | 0.2161 |
| 130 | Long Gu | Wxa | 0.2372 |
| 131 | Lu Shui Nuo | Wxa | 0.2206 |
| 132 | Luan Jiao Bai Gu | Wxa | 0.2296 |
| 133 | Luan Jiao Diao | Wxa | 0.2141 |
| 134 | Lue Ha | Wxa | 0.2397 |
| 135 | Luo Ping Gu | Wxa | 0.2236 |
| 136 | Ma Deng Hong Gu | Wxa | 0.2281 |
| 137 | Ma Ti Gu | Wxa | 0.2342 |
| 138 | Ma Xian Gu(1)* | Wxa | 0.2562 |
| 139 | Ma Xian Gu(2)* | Wxb | 0.0374 |
| 140 | Ma Zha Gu(1)* | Wxb | 0.2733 |
| 141 | Ma Zha Gu(2)* | Wxa | 0.2382 |
| 142 | Ma Zha Gu(3)* | Wxa | 0.2552 |
| 143 | Ma Zha Gu(4)* | Wxa | 0.2967 |
| 144 | Ma Zha Gu(5)* | Wxa | 0.2648 |
| 145 | Ma Zha Nuo | Wxa | 0.2201 |
| 146 | Mang Shi Gu | Wxa | 0.2648 |
| 147 | Mang Zhong Gu | Wxa | 0.2939 |
| 148 | Mao Ke Hong Nuo | Wxa | 0.2402 |
| 149 | Mao Xiang Gu | Wxa | 0.2332 |
| 150 | Mao Xiang Nuo | Wxa | 0.2628 |

| | | | |
|---|---|---|---|
| 151 | Meng Ka Zha | Wxa | 0.2166 |
| 152 | Meng Lu Gu | Wxa | 0.2753 |
| 153 | Meng Wang Gu(1)* | Wxb | 0.1594 |
| 154 | Meng Wang Gu(2)* | Wxmw | 0.123 |
| 155 | Meng Xian Da Bai Gu | Wxa | 0.2593 |
| 156 | Mo Wang Gu | Wxa | 0.2276 |
| 157 | Niao Ju Qi Tou | Wxa | 0.2487 |
| 158 | Niu Yang Gu | Wxa | 0.2618 |
| 159 | Ou Yang Gu | Wxa | 0.2803 |
| 160 | Qi Tou Gu | Wxa | 0.2467 |
| 161 | Qi Yue Nuo | Wxa | 0.1955 |
| 162 | Qing Ming Li Hong Gu | Wxa | 0.2613 |
| 163 | Shan Dao Hong Gu | Wxa | 0.2703 |
| 164 | Ta Pi Gu | Wxa | 0.1257 |
| 165 | Tu Po Huo Lue | Wxa | 0.191 |
| 166 | Tuan Ke Li | Wxa | 0.2487 |
| 167 | Wu Bai Li | Wxa | 0.201 |
| 168 | Wu Ju Gu | Wxa | 0.2567 |
| 169 | Xi Bai Gu(1)* | Wxa | 0.2673 |
| 170 | Xi Bai Gu(2)* | Wxa | 0.2322 |
| 171 | Xi Gan Qi Tou | Wxa | 0.2603 |
| 172 | Xi Luo Ping | Wxa | 0.2301 |
| 173 | Xi Ma Xian Gu | Wxa | 0.2101 |
| 174 | Xi Ma Zha Gu | Wxb | 0.1132 |
| 175 | Xiang Diao | Wxa | 0.2628 |
| 176 | Xiang Gu(1)* | Wxa | 0.2829 |
| 177 | Xiang Gu(2)* | Wxa | 0.2633 |
| 178 | Xiang Gu(3)* | Wxa | 0.2437 |
| 179 | Xiang Nuo | Wxa | 0.2209 |
| 180 | Xiao Bai Gu | Wxa | 0.2628 |
| 181 | Xiao Bai Nuo | Wxa | 0.2547 |
| 182 | Xiao Hong Gu(1)* | Wxa | 0.1935 |
| 183 | Xiao Hong Gu(2)* | Wxa | 0.2291 |
| 184 | Xiao Hong Gu(3)* | Wxa | 0.2035 |
| 185 | Xiao Luo Ping | Wxa | 0.2638 |
| 186 | Xiao Ma Zha Gu | Wxin | 0.2517 |
| 187 | Xiao Wu Ju Gu | Wxa | 0.2608 |
| 188 | Xiao Zi Gan | Wxa | 0.2382 |
| 189 | Xin Tuan Hei Gu | Wxin | 0.2241 |
| 190 | Xiu Shui Gu | Wxa | 0.2638 |
| 191 | Yi Liang Xian | Wxa | 0.2658 |
| 192 | Yuan Jiang Bai Gu | Wxa | 0.1794 |
| 193 | Yuan Jiang Zao Gu | Wxa | 0.2492 |
| 194 | Yue Liang Gu | Wxa | 0.2673 |

| | | | |
|---|---|---|---|
| 195 | Yun Xian Hong Mi | Wxa | 0.0951 |
| 196 | Zao Diao | Wxa | 0.1985 |
| 197 | Zao Er Kuai | Wxa | 0.2322 |
| 198 | Zao Gu | Wxa | 0.2276 |
| 199 | Zi Gan Gu | Wxa | 0.2527 |
| 200 | Zi Gu | Wxin | 0.2136 |
| 201 | Zi Nuo | Wxa | 0.1609 |

*Yunnan Local Rice Germplasm Accessions Sharing Identical Names but Originating from Different Sources.
